# Supplementary material for: High-Frequency Pulsed Electric Field Ablation in Beagle Model for Treatment of Prostate Cancer
Source: Cancers (Basel). 2022 Oct 12;14(20):4987. doi: 10.3390/cancers14204987 (PMC9599345; doi:10.3390/cancers14204987)
Supplement: Supplementary file 1 [file cancers-14-04987-s001.zip › cancers-1910132-SI.pdf]

cancers

MDPI

Supplementary Material

High-Frequency Pulsed Electric Field Ablation in Beagle Model for treatment of Prostate Cancer

Seung Jeong[1†], Song Hee Kim[2†], Hongbae Kim[1], Jeon Min Kang[2], Yubeen Park[2], Dong-Sung Won[2], Ji Won Kim[2], Dae Sung Ryu[2], Chu Hui Zeng[2], Jong Hoon Chung[1,3], Bumjin Lim[4]*, Jung-Hoon Park[2*]

[1] Department of Biosystems & Biomaterials Science and Engineering, Seoul National University, Seoul, 08826, Republic of Korea

[2] Biomedical Engineering Research Center, Asan Institute for Life Sciences, Asan Medical Center, 88 Olympic-ro 43-gil, Songpa-gu, Seoul, 05505, Republic of Korea

[3] Research Institute for Agriculture and Life Sciences, Seoul National University, Seoul, 08826, Republic of Korea

[4] Department of Urology, Asan Medical Center, University of Ulsan College of Medicine, 88 Olympic-ro 43-gil, Songpa-gu, Seoul, 05505, Republic of Korea

[†]These authors have contributed equally to this work and share first authorship

*** Correspondence:**
Bumjin Lim M.D.[1], and Jung-Hoon Park, Ph.D.[2]
[1]Department of Urology, Asan Medical Center, University of Ulsan College of Medicine, 88 Olympic-ro 43-gil, Songpa-gu, Seoul 05505, Republic of Korea
Tel: 82-2-3010-1835 Fax: 82-2-476-0090
E-mail: lbj1986@hanmail.net
[2]Biomedical Engineering Research Center, Asan Institute for Life Sciences, Asan Medical Center, 88 Olympic-ro 43-gil, Songpa-gu, Seoul, 05505, Republic of Korea
Tel: 82-2-3010-4123 Fax: 82-2-476-0090
E-mail: jhparkz@amc.seoul.kr

Keywords: Irreversible electroporation, High-frequency pulsed electric field, Prostate Cancer, Decellularization, Regeneration

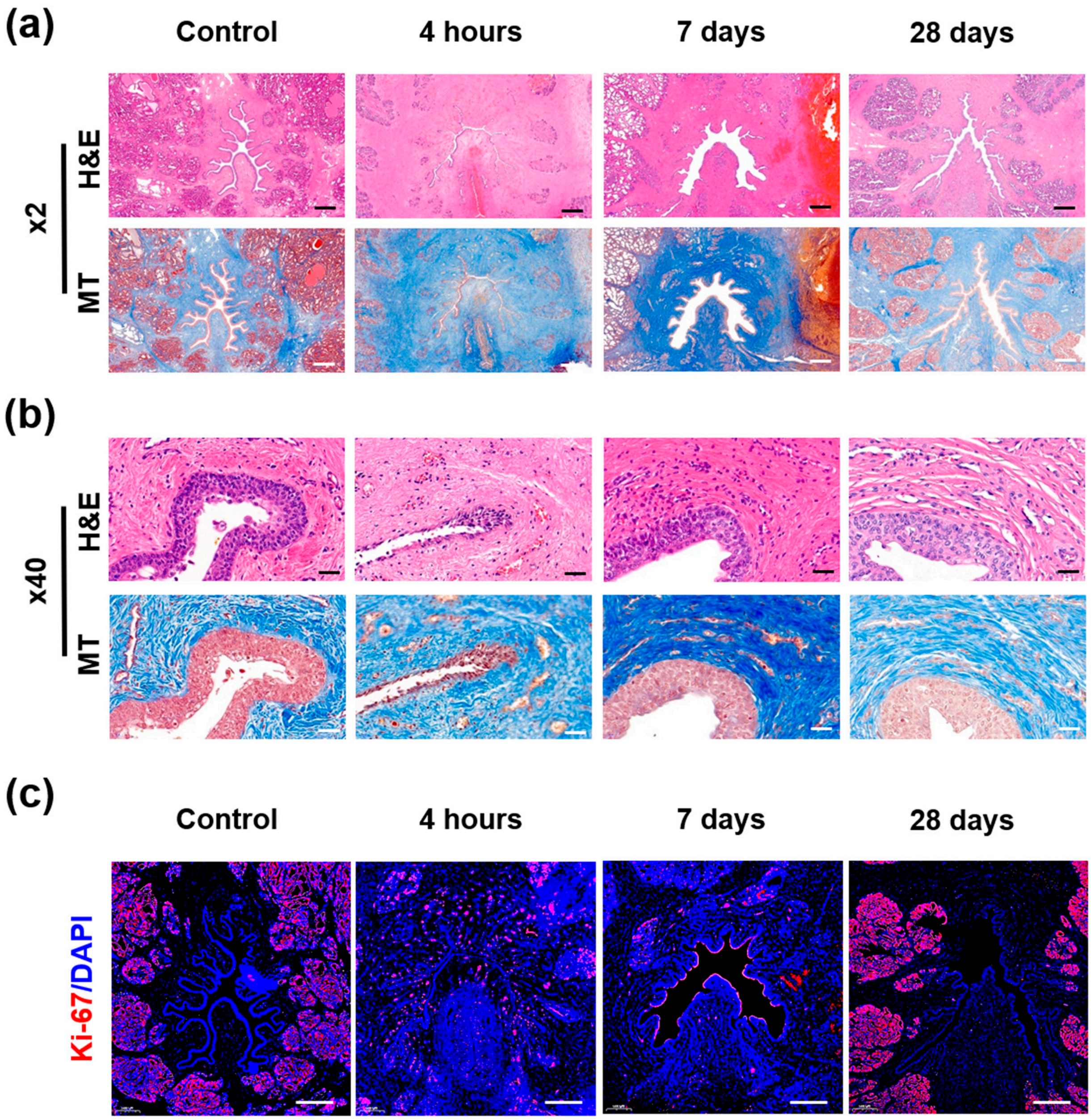

Supplementary Figure S1. Histological findings of the prostate after irreversible electroporation (IRE) with high-frequency pulsed electric field (HF-PEF). Hematoxylin and eosin (H&E), Masson's trichrome (MT), and terminal deoxynucleotidyl transferase-mediated dUTP nick and labeling (TUNEL)-stained sections after IRE with HF-PEF showed gradual histological changes.

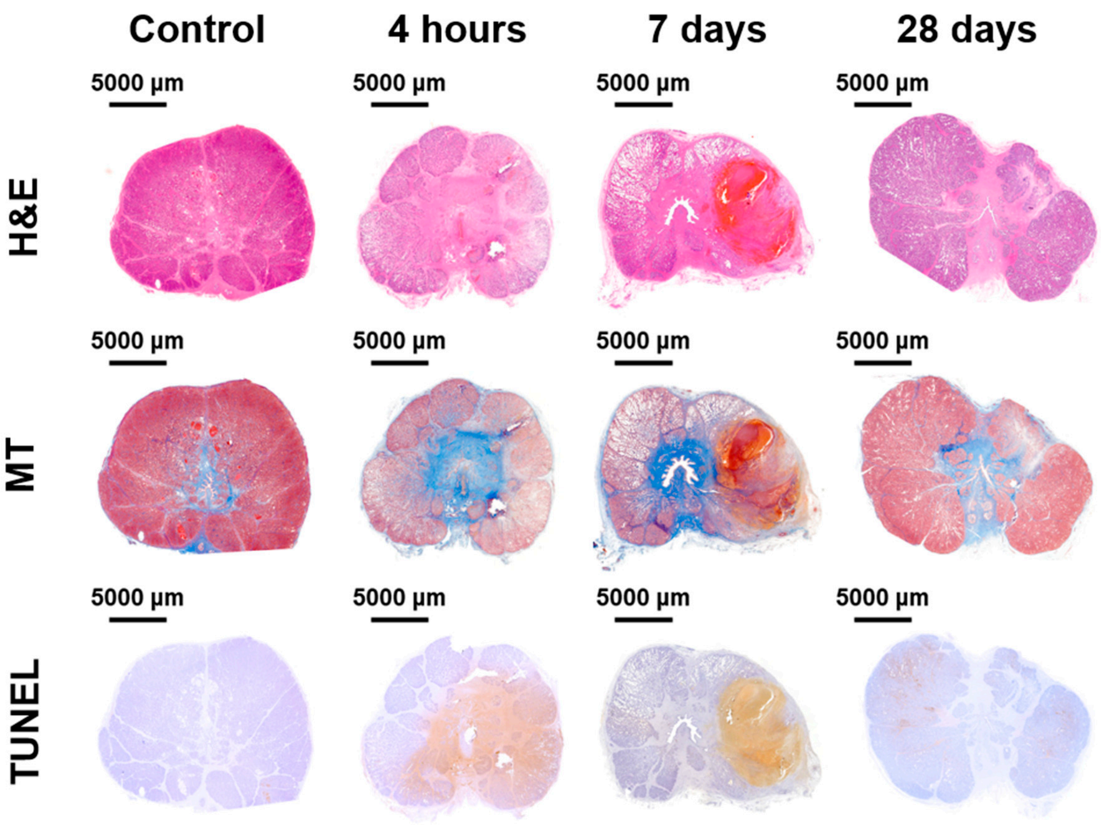

Supplementary Figure S2. Representative microscopic images of histopathological sections around the urethra. Hematoxylin and eosin (H&E)- and Masson's trichrome (MT)-stained sections are shown at (a) 2× magnification (scale bar: 5 mm) and (b) 40× magnification (scale bar: 250 µm). (c) Ki-67 immunofluorescence (DAPI, blue; and Ki67, red) images showing active cellular regeneration around the urethra (scale bar, 1 mm; 2× magnification).

Supplementary Table S1. Histological findings after IRE with HF-PEF in the beagle prostate

	Control	4 hours	*p*-value	7 days	*p*-value	28 days	*p*-value
**Inflammatory cell infiltration (grade)**	1.11 ± 0.33	1.40 ± 0.52	0.164	4.00 ± 1.12	0.014	2.80 ± 0.60	0.029
**MT fibrotic area (mm^2)**	21.64 ± 0.58	66.75 ± 14.03	0.030	32.04 ± 5.10	0.070	30.31 ± 14.34	0.145
**TUNEL ablation area (mm^2)**	2.69 ± 0.51	113.53 ± 5.57	0.003	41.65 ± 5.99	0.015	4.46 ± 1.20	0.063
**Ki-67 Positive area (%)**	0.70 ± 0.67	0.14 ±0.22	0.017	0.51 ± 0.13	0.090	0.57 ± 0.07	0.082

Note. Data are presented as means ± standard deviations
